# Crop root system plasticity for improved yields in saline soils

**DOI:** 10.3389/fpls.2023.1120583

**Published:** 2023-02-24

**Authors:** Megan C. Shelden, Rana Munns

**Affiliations:** ^1^ School of Agriculture, Food and Wine, University of Adelaide, Urrbrae, SA, Australia; ^2^ Australian Research Council (ARC) Centre of Excellence in Plant Energy Biology, School of Molecular Sciences, University of Western Australia, Crawley, WA, Australia

**Keywords:** salinity, root elongation, root system architecture, abiotic stress, wheat, barley, rice, crops

## Abstract

Crop yields must increase to meet the demands of a growing world population. Soil salinization is increasing due to the impacts of climate change, reducing the area of arable land for crop production. Plant root systems are plastic, and their architecture can be modulated to (1) acquire nutrients and water for growth, and (2) respond to hostile soil environments. Saline soils inhibit primary root growth and alter root system architecture (RSA) of crop plants. In this review, we explore how crop root systems respond and adapt to salinity, focusing predominately on the staple cereal crops wheat, maize, rice, and barley, that all play a major role in global food security. Cereal crops are classified as glycophytes (salt-sensitive) however salt-tolerance can differ both between species and within a species. In the past, due to the inherent difficulties associated with visualising and measuring root traits, crop breeding strategies have tended to focus on optimising shoot traits. High-resolution phenotyping techniques now make it possible to visualise and measure root traits in soil systems. A *steep, deep and cheap* root ideotype has been proposed for water and nitrogen capture. Changes in RSA can be an adaptive strategy to avoid saline soils whilst optimising nutrient and water acquisition. In this review we propose a new model for designing crops with a salt-tolerant root ideotype. The proposed root ideotype would exhibit root plasticity to adapt to saline soils, root anatomical changes to conserve energy and restrict sodium (Na^+^) uptake, and transport mechanisms to reduce the amount of Na^+^ transported to leaves. In the future, combining high-resolution root phenotyping with advances in crop genetics will allow us to uncover root traits in complex crop species such as wheat, that can be incorporated into crop breeding programs for yield stability in saline soils.

## 1 Introduction

Increasing soil salinization decreases plant growth and impacts crop productivity world-wide. Of particular concern is the salinization of irrigated land, as this provides nearly half of the world’s food (Hopmans et al., 2021). An increase in the global demand for food for a growing population means that crops will increasingly be grown on salt-affected soils. Saline soils are defined as having an electrical conductivity (EC) greater than 4 dS m^-1^ (40 mM NaCl), which can lead to a reduction in crop growth of 15 – 20% (Munns and Tester, 2008). However, saline soils can be much higher than this; salt distribution in soils is never uniform across an area and will vary with depth making it difficult to precisely calculate crop yield losses (Munns et al., 2020a). Glycophytes are salt sensitive species, tolerating only low concentrations of salt and include our most staple food crops (ie. wheat, maize, rice and barley). Halophytes are highly salt tolerant, growing in NaCl concentrations up to 500 mM. Compared with glycophytic plants they can accumulate high concentrations of salt (Na^+^ and Cl^-^) in their leaf tissues to balance water relations (Colmer et al., 2006). Salt tolerance varies in glycophytic species, with barley and wheat moderately salt-tolerant compared to both Arabidopsis and rice (Munns and Tester, 2008). Salt tolerance can also vary within a species.

In terms of global food production, the cereal crops wheat (*Triticum* sp. L.), maize (*Zea mays* L.), rice (*Oryza sativa* L.) and barley (*Hordeum vulgare* L.) are the four most important crops. Wheat, grown in temperate regions, is the largest food crop in the world, providing approximately 20% of human calories and protein (Tadesse et al., 2019). Maize is grown predominantly in the Americas for grain, fodder, and as raw materials for industry (production of biofuels) (Shiferaw et al., 2011). Rice is a dietary staple for more than half of the world’s population. The Global Rice Science Partnership is predicting a 26% increase in rice production will be required in the next 25 years to meet global food demands (Zeigler and Dobermann, 2011). Together rice, wheat, and maize account for more than 50% human calorific consumption (FAO, 2022) and this is closer to 60% for developed countries (Awika, 2011). Barley is the fourth most important crop and is primarily grown for malting and livestock feed. It is the most salt-tolerant species out of the cereal crops, followed by wheat (moderately-tolerant), maize (moderately-sensitive) and rice (sensitive) (Munns and Tester, 2008), and is therefore an excellent model species for studying salt-tolerance mechanisms in crop plants.

Climate change is likely to accelerate soil salinization, because of the increased crop water requirements by elevated temperatures, through sea level rise, and the reduction in freshwater available for irrigation (Hopmans et al., 2021). The global impact of the changing climate is seen in interactions and feedbacks between climate, land degradation and food security. A significant increase in productivity of all cereal crops on salinized land is needed.

Plant roots have a pivotal role in providing anchorage, as well as the uptake of water and nutrients from the soil, for the maintenance of plant growth. The roots adapt to changes in the environment and soil conditions through many mechanisms, including the activity of membrane transporters that control the uptake of ions and water from the soil, their transport through the roots, and the loading into the xylem (Schroeder et al., 2013). In response to saline soils, the roots must exclude nearly all the salt from the soil solution (97 - 98%) whilst continuing to take up water by transpiration (Munns et al., 2020b). Plants exhibit root plasticity and can modulate their root system architecture (RSA) in response to their environment including hostile soils ie. drought, salt, flooding and extreme temperatures (Karlova et al., 2021). RSA also responds to soil nutrient deficiencies including nitrogen (N) and phosphorous (P) (Lynch, 2019). The *steep, cheap and deep* root ideotype, consists of physiological, anatomical and architecture traits for improved N acquisition in maize (Lynch, 2019).

Root traits have been studied less than shoot traits, mainly due to the inherent difficulties of imaging and quantifying root growth in soils. Modern root phenotyping technology has led to significant advancements in selecting root traits for improved crop productivity with promising root traits selected for abiotic stress tolerance incorporated into new germplasm for breeders. These include narrow xylem vessels to conserve water, shallow roots for surface P uptake, and longer roots for deep water capture (Tracy et al., 2020). Soil-surface roots (SOR) may also be beneficial for waterlogging tolerance in upland species such as rice to avoid hypoxic conditions (Hanzawa et al., 2013). Complex traits such as salinity tolerance are multigenic, therefore it is difficult to identify candidate genes for incorporation into breeding programs. Exploiting natural genetic diversity within a crop species by screening varieties, wild genotypes and landraces will be key for breeding more salt tolerant crops in the future (Shelden and Roessner, 2013). Genes for exclusion of Na^+^ from the xylem stream flowing to leaves in saline soil have been transferred into roots of modern wheat (Munns et al., 2012), but no specific trait for RSA that enhance salt-tolerance have been reported.

In this review we highlight the mechanisms that control crop root growth and development in response to salinity. We explore how this knowledge can be used to improve root system architecture for crop growth and yield in saline conditions. With significant gains in high resolution spatial profiling technologies and phenotyping, these tools can be used to understand salt-tolerance mechanisms from the individual cell to the whole plant system. The discovery of traits for root system adaptation to salinity stress will provide an under-utilised trait for breeders to develop more salt-tolerant crops.

## 2 Structure of crop root systems

**Figure 1:**
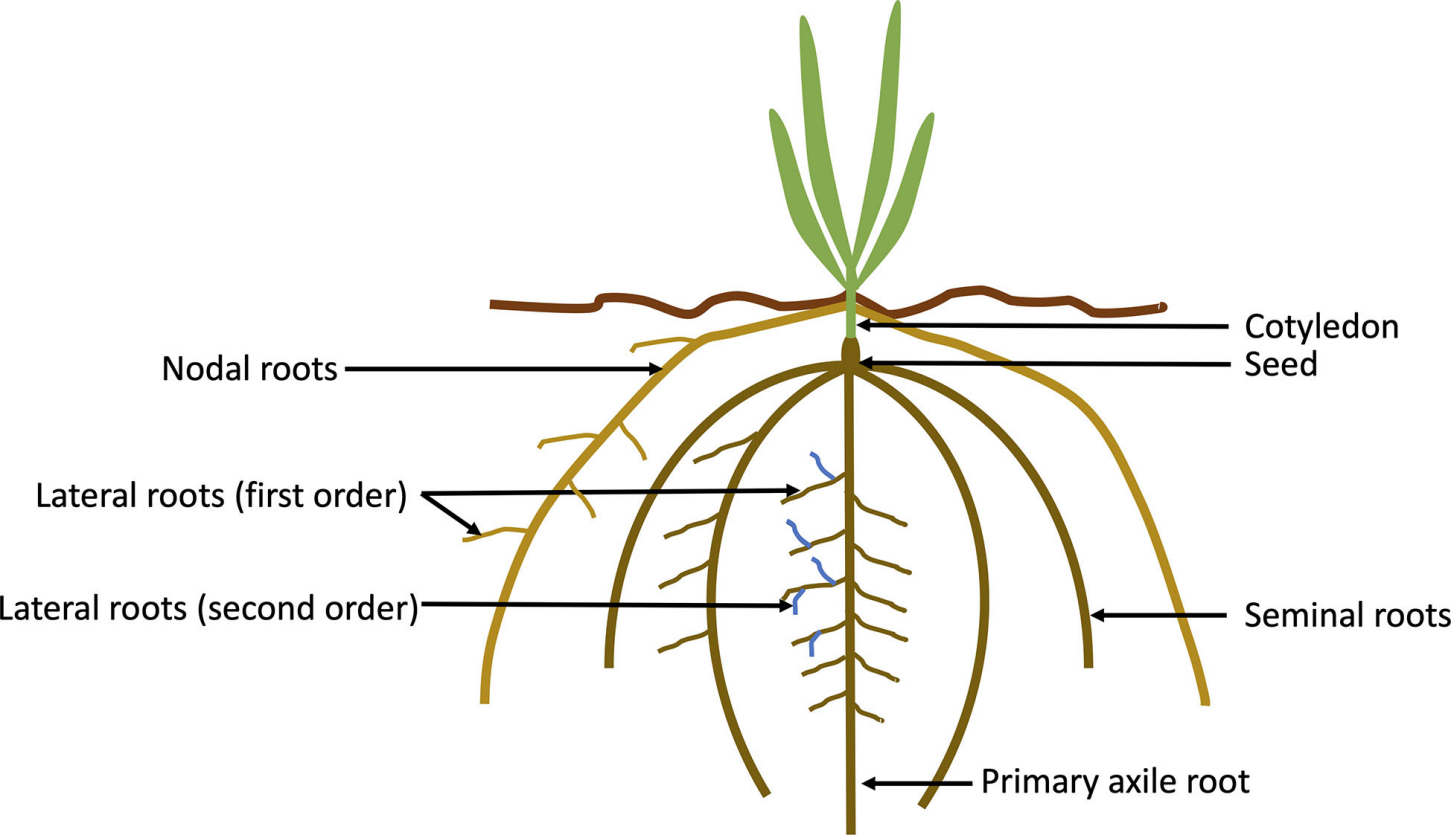
Schematic drawing of the components of a cereal (monocotyledon) root system. Cereal crops have a fibrous root system with primary and seminal embryonic roots (numbers can differ between species), lateral roots (also known as branch roots), and nodal roots.

Cereal crops are monocots, and their root structure comprises both embryonic (primary and seminal) and post-embryonic (brace, nodal and lateral) roots (**Figure 1**). Monocots and dicots are characterised by different root structures, with dicots having an embryonic primary root (PR), lateral roots (LR) and adventitious roots (AR) (Smith and De Smet, 2012). Monocots have a fibrous root system and shoot borne roots that overtake the embryonic PR and LR and are mostly responsible for water and nutrient uptake. Barley, wheat, and maize have a variable number of seminal roots (including the primary root), with up to seven in some barley cultivars (Bengough et al., 2004), however this number can vary within species, and can also be affected by abiotic stresses. In contrast, the rice root system is comprised of a single embryonic seminal root, crown, and lateral roots. In maize and sorghum, below-borne roots are often referred to as crown roots and above ground brace (or prop) roots (Hochholdinger et al., 2004; Maqbool et al., 2022). In other cereals such as barley and wheat the shoot-borne roots are referred to as nodal roots. In cereals, the seminal root system is only active early in the growing period (up to the 4-leaf stage in barley and wheat).

Root hairs are specialised tubular structures that arise from epidermal cells. They are crucial for nutrient and water capture as they extend into the soil and increase the root surface area. The growth and development of root hairs are modulated by environmental signals, ensuring optimal acquisition of nutrients. The availability of less mobile nutrients such as inorganic P (Pi), iron (Fe), zinc (Zn) and magnesium (Mg) can all affect root hair morphogenesis (Lynch et al., 2014; Salazar-Henao et al., 2016). Root hairs are also important for the development and stabilization of the rhizosphere (McCully, 1999), and they can enhance penetration of high-strength soils (Haling et al., 2013). Root hair growth and development can also be affected by abiotic stresses, with saline soils shown to decrease both root hair length and density (Wang et al., 2008; Robin et al., 2016). In barley, root hairs also have been shown to contribute to drought tolerance and improved yield stability (Marin et al., 2020). Root hairs form part of the rhizosheath, along with soil particles bound to root hairs, mucilage and the root microbiome (McCully, 1999). Salt stress can influence the constitution of soil bacterial communities (Rath et al., 2019). Understanding the impact of saline soils on root-associated microbial communities will be important in generating crops with root systems adapted to salinity (Qin et al., 2016).

## 3 Primary and seminal root growth inhibition in response to salinity

Salinity inhibits root system growth less than shoot growth, and the root:shoot biomass ratio increases in most species including sorghum (Weimberg et al., 1984), wheat (Husain et al., 2004), and rice (Lin and Kao, 1996). At low salinity, root system growth may not decrease at all while shoot growth declines as in barley and sorghum, or it may even increase as in bermudagrass and Cynodon turf grass (reviewed by Munns and Termaat (1986)). This may be due to stimulation of the rate of individual root elongation, as reported for cotton (*Gossypium hirsutum* L.) plants in saline solution by Kurth et al. (1986). In both barley and wheat, genetic variation has been observed in the primary root response to salinity (Rahnama et al., 2011; Shelden et al., 2013).

Elongation rate of the primary root was reduced by salinity up to 150 mM NaCl in *Arabidopsis* (Wu et al., 1996), barley (Shelden et al., 2013), wheat (Rahnama et al., 2011) and maize (Bernstein and Kafkafi, 2002). However, in cotton seedlings, the growth of the primary root was enhanced by moderate salinities (25 to 100 mM NaCl), and the cortical cells were longer than in control plants (Zhong and Lauchli, 1993). This increase occurred only when the Ca^2+^ concentration in the solution was supplied at 10 mM Ca^2+^ (Zhong and Lauchli, 1993). The rate of cell production in the presence of 10 mM Ca^2+^ was not affected by salinities up to 150 mM NaCl (Kurth et al., 1986). These findings reinforce the importance of having sufficient Ca(NO_3_)_2_ in the medium to prevent the Ca^2+^ activity falling in the presence of NaCl. The addition of NaCl or any electrolyte decreases the activity (the concentration of the free Ca^2+^ ion). For instance, addition of 150 mM NaCl to a solution containing 2 mM Ca(NO_3_)_2_ (i.e. half-strength Hoagland solution) decreases the activity of free Ca^2+^ to 0.64 mM (Husain et al., 2004). Raising the Ca^2+^ activity from 0.64 to 3.15 mM by increasing the Ca(NO_3_)_2_ from 2 to 10 mM in the presence of 150 mM NaCl resulted in a 50% increase in root system biomass in wheat (Husain et al., 2004). Calcium is an essential nutrient. It is a constituent of cell walls and affects many membrane functions including membrane permeability to cations such as Na^+^ (White and Broadley, 2003). The Ca^2+^ activity should be close to 1 mM (Genc et al., 2010). The Ca^2+^ concentration in irrigation water and groundwater of saline soils is higher than this (Rengasamy, 2010); salt treatments in laboratory experiments should replicate the natural environment when possible.

### 3.1 Root growing zone – cell division versus cell elongation

**Figure 2:**
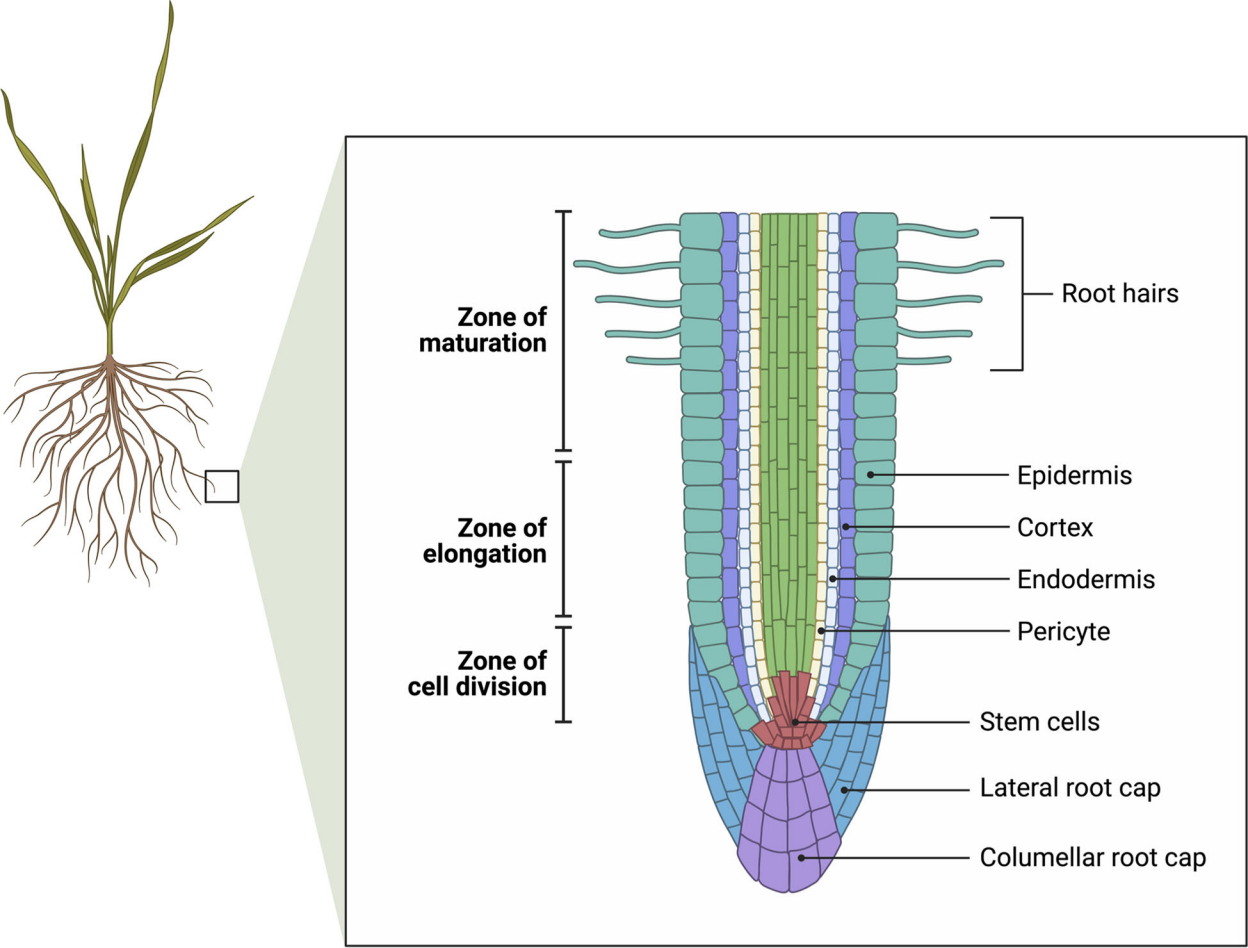
Longitudinal view of root tip showing the zones of cell division (the meristem), elongation, and maturation. Cell division defines the apical meristem. Root hairs begin to develop in the maturation zone. Adapted from “Root Meristem Anatomy” by BioRender.com (2022). Retrieved from https://app.biorender.com/biorender-templates.

Root growth is limited to a small region of the root tip comprising the root apical meristem, cell division zone and elongation zone (**Figure 2**). In barley and rye (*Secale cereale* L.), the meristematic zone extends to about 1 mm from the tip, and the elongating zone another 2 mm from the tip (Ogawa et al., 2006, Shelden et al., 2016). In rye, moderate salt stress up to 100 mM NaCl did not inhibit cell division nor the elongation rate of the root (note that Ca(NO_3_)_2_ was supplied at 4 mM which would be sufficient to maintain the Ca^2+^ activity above 1 mM), but at 250 mM NaCl cell division ceased (Ogawa et al., 2006). In barley cv. Clipper the length of the cell division zone was unaffected by salt, however the elongation zone was shorter accounting for a reduced elongation rate (Shelden et al., 2016). In contrast, in the barley landrace Sahara 3771, cell division was inhibited and was primarily responsible for the reduction in root elongation rate (Shelden et al., 2016). In Arabidopsis, salinity results in an inhibition of cell production, cell cycle progression and a reduction in meristem size (West et al., 2004). Kinematic analysis revealed that the growth reduction of the stressed roots (83 mM NaCl) was due to a decrease in cell production and a smaller mature cell length. The average cell cycle duration was not affected (West et al., 2004). In cotton and maize, the length of the growing zones is intrinsically longer, up to 10 mm from the tip (Zhong and Lauchli, 1993 for cotton) (Rodriguez et al., 1997 for maize). Kinematic analysis showed that the decrease in root growth is due to an inhibition of both cell division and elongation (Zhong and Lauchli, 1993; Bernstein and Kafkafi, 2002). The length of the elongation zone is shortened in response to salinity in maize (Zidan et al., 1990), cotton (Zhong and Lauchli, 1993), barley (Shelden et al., 2016) and sorghum (*Sorghum bicolor* L.) (Koyro, 1997).

### 3.2 Osmotic and ionic stress

The ionic (salt-specific) effect of external concentrations of Na^+^ and Cl^-^ on root growth is considered secondary in time to the initial osmotic effect of salinity, which cause the same rapid decrease in root growth as do non-ionic osmotica such as mannitol and polyethylene glycol. That is, the initial effects are from the salt outside the roots, not on the inside (Munns, 2002). High concentrations of Na^+^ and Cl^-^ ions in the soil make it more ‘difficult’ (that is, require more energy) for roots to extract water, resulting in an ‘osmotic stress’ similar to that occurring with water deficit (Rodriguez et al., 1997; Yamaguchi and Sharp, 2010).

In barley, root elongation rate decreased within hours of exposure to NaCl, suggesting that the initial effect of NaCl on the root is due to an osmotic stress (Shelden et al., 2013). After three days growth on 150 mM NaCl, root and shoot Na^+^ concentrations increased up to 17-fold compared to control conditions and the K^+^/Na^+^ ratio significantly decreased. However, root (and shoot) ion concentrations did not correlate with root elongation rates, showing that the Na^+^ and K^+^ concentrations in the mature zones of roots were not influencing root growth, at least during the early phase of salt stress (Shelden et al., 2013). In maize plants treated with high concentrations of NaCl in a single step ‘shock’ treatment, there was a rapid decrease in root growth, however this was not seen in a salt acclimation treatment when the NaCl was increased incrementally (Rodriguez et al., 1997), also suggesting osmotic stress is important. A link between root growth and root ion content was also reported in tomato (*Solanum lycopersicum L.*); the most salt-sensitive genotypes (with respect to root growth) had the highest root Na^+^ content and lowest K^+^/Na^+^ ratio (Gandullo et al., 2021). This contrasts with barley, where no correlation between root growth and K^+^/Na^+^ ratio was observed (Shelden et al., 2013).

Ion concentrations in plants subject to salinity stress have routinely been measured in whole plant tissues (roots, leaves, and even whole shoots). To understand the direct impact of cellular Na^+^ concentration on root cell division and elongation, high resolution spatial profiling of root ion concentrations is needed, as done using quantitative cryo-analytical scanning electron microscopy with X-ray microanalysis in wheat roots (Lauchli et al., 2008) and in barley roots (Kotula et al., 2015). Differences in cellular root Na^+^ were reported in aerated saline conditions in barley at 10 mm and 50 mm behind the root apex (Kotula et al., 2015). Closer to the root tip, Na^+^ was higher in the outer tissues (epidermis, outer cortex) and lower in the inner cells and xylem vessels. In contrast, at 50 mm from the apex, Na^+^ and Cl^-^ were high in the pericycle and lower in the outer tissues.

The fluorescent dye CoroNa green has been used to determine intracellular cytosol and vacuolar Na^+^ however as the dye is non-ratio metric measurements cannot by quantified (Park et al., 2009; Wu et al., 2015). In barley, laser ablation inductively coupled proton mass spectrometry (LA-ICP-MS) was used to show that Na^+^ concentrations in the root meristem and elongation zone remained relatively low when plants were exposed to salt stress (Shelden et al., 2020). This indicated that in the moderately salt-tolerant crop barley, internal cellular concentrations of Na^+^ in the meristem do not directly inhibit cell division; instead, as in water stress, cell division may be regulated by a process involving abscisic acid and the properties of the expanding primary cell wall (Yamaguchi and Sharp, 2010). A number of studies have shown the ability of glycophytes to exclude Na^+^, but not Cl^-^, from the root meristem including in maize (Rodriguez et al., 1997).

Na^+^ and Cl^-^ is taken up from the soil into the epidermis, transported across the root and loaded into the xylem *via* membrane transporters. The active removal of Na^+^ and Cl^-^ from the cytoplasm into the vacuoles allows cells and tissues to tolerate higher internal concentrations of Na^+^ (tissue tolerance). In most higher plants including crop species, Na^+^ appears to reach toxic concentrations before Cl^-^ and therefore is thought to be more damaging to the plant (Munns and Tester, 2008). In some species such as citrus (*Citrus* sp.) and grapevine (*Vitis vinifera* L*.)*, Cl^-^ concentrations in the leaf blades rise faster than Na^+^ (Na^+^ is retained in stems and petioles) and so Cl^-^ is considered as the more toxic ion (Storey and Walker, 1998; Zhou-Tsang et al., 2021). Loading of Cl^-^ into the vacuole of root cortical cells plays a role in Cl^-^ exclusion from the shoots (Teakle and Tyerman, 2010). Salt-treatment of plants also affects the cellular concentrations of other ions. The uptake of potassium (K^+^) is reduced which has potentially negative effects on cellular metabolism and signalling (Wu et al., 2018b).

In durum wheat grown at 50 mM NaCl, X-ray microanalysis of mature roots showed that the Na^+^ concentration declined across the cortex, being highest in the epidermal and subepidermal cells (48 mM) and lowest in the inner cortical cells (22 mM) (Lauchli et al., 2008). Na^+^ was high in the pericycle (85 or 150 mM, depending on genotype) and low in the xylem parenchyma (34 mM). The K^+^ profiles were generally inverse to those of Na^+^. Chloride was only detected in the epidermis. These data suggest that the epidermal and cortical cells removed most of the Na^+^ and Cl^-^ from the transpiration stream before it reached the endodermis, and that the endodermis is not the control point for salt uptake by the plant. The pericycle as well as the xylem parenchyma may be important in the control of net Na^+^ loading of the xylem, their vacuoles acting as sites for storage.

Osmotic adjustment is an essential adaptation to saline soil. Plants growing in saline soils regulate the uptake of Na^+^ and Cl^-^ to avoid ion toxicity, while ensuring sufficient solutes for osmotic adjustment. Root cells accumulate sufficient solutes to match, in osmolarity, the increased ion concentrations in the soil solution. This ‘osmotic adjustment’ maintains cell turgor and the volume of organelles within the cells of the growing plant. Taking up just 2% of the NaCl allows a plant to osmotically adjust the Na^+^ and Cl^–^ in vacuoles, while organic solutes provide the balancing osmotic pressure in the cytoplasm (Munns et al., 2020b). Osmotic adjustment through the synthesis of compatible solutes in roots has been observed in maize (Rodriguez et al., 1997) and barley (Shelden et al., 2016). The maintenance of cell division and root elongation in barley in response to salt stress, was linked to the synthesis of compatible solutes for osmotic adjustment and restoration of cell turgor (Shelden et al., 2016).

## 4 Plant roots sense changes in osmolarity and sodium

How plant roots “sense” Na^+^ in the soil and initiate the salt-specific response is still unknown. In animals, distinct protein receptors have been shown to be involved in sensing salt, with several ion channels found to act as salt sensing taste receptors (Chatzigeorgiou et al., 2013; Zhang et al., 2013). In roots, the sensor may be external to the epidermal cells, and detected by the proteins or lipids on the cell membrane. Alternatively, it could be within the cell and respond to small increases in cytosolic Na^+^. It is also assumed that plants must have mechanisms to detect both osmotic (water deficit) and ionic (salt-specific) stress. However, limited progress has been made because of the common usage of ‘salt shock’, a transfer of plants growing in non-saline hydroponic solutions to very high concentrations of NaCl or mannitol. This immediately causes a very large but transient rise in the concentration of cytosolic Ca^2+^. In Arabidopsis, OSCA1 is a hyperosmolality-gated calcium-permeable channel that results in increased cytosolic Ca^2+^ (Yuan et al., 2014). Structural analysis of OSCA family members have shown these belong to a mechanosensitive ion channel. In rice, cryo-electron microscopy (Cryo-EM) structure and function have shown how the rice homolog OSCA1 can mediate hyperosmolality sensing and transport pathway gating (Maity et al., 2019). Homologs of OSCA1 have been identified in the genome of rice (Li et al., 2015), maize (Cao et al., 2020), wheat (Tong et al., 2022) and soybean (Liu et al., 2022a). However, the role of OSCA1 as an ‘osmosensor’ is not yet proven as most experiments were done with very high concentrations of NaCl or mannitol which would cause plasmolysis, that is, extensive shrinkage of the protoplast with stretching of the plasma membrane between the sites where it remains tethered to the cell walls (Munns, 2002).

Salt stress sensors include both osmotic stress and Na^+^ sensors and may include pectin in cell walls, ion transporters, mechanosensory proteins, purine receptors, annexins and voltage-gated channels (Shabala et al., 2015; Wang et al., 2022). In the model plant Arabidopsis*, AtIPUT1* that encodes an inositol phosphorylceramide glucuronosyltransferase, is required for the synthesis of glycosyl inositol phosphorylceramide (GIPC) sphingolipids, that is proposed as a potential salt sensor (Jiang et al., 2019). GIPCs are a class of glycosylated sphingolipids that are highly enriched in the outer leaflet of the plasma membrane (Mortimer and Scheller, 2020). GIPCs have been detected in barley using lipodomics (Yu et al., 2018), and in cotton, GIPC profiles change in response to salinity (Liu et al., 2022c). GIPCs are anchored in the plasma membrane and are activated by Ca^2+^ influx, leading to the up-regulation of Na^+^/H^+^ antiporters. Overexpression of *IbIPUT1* in sweet potato (*Ipomoea batatas* (L.) Lam), resulted in a decrease in the accumulation of Na^+^ in root cells in response to salt stress (Liu et al., 2022b).

**Figure 3:**
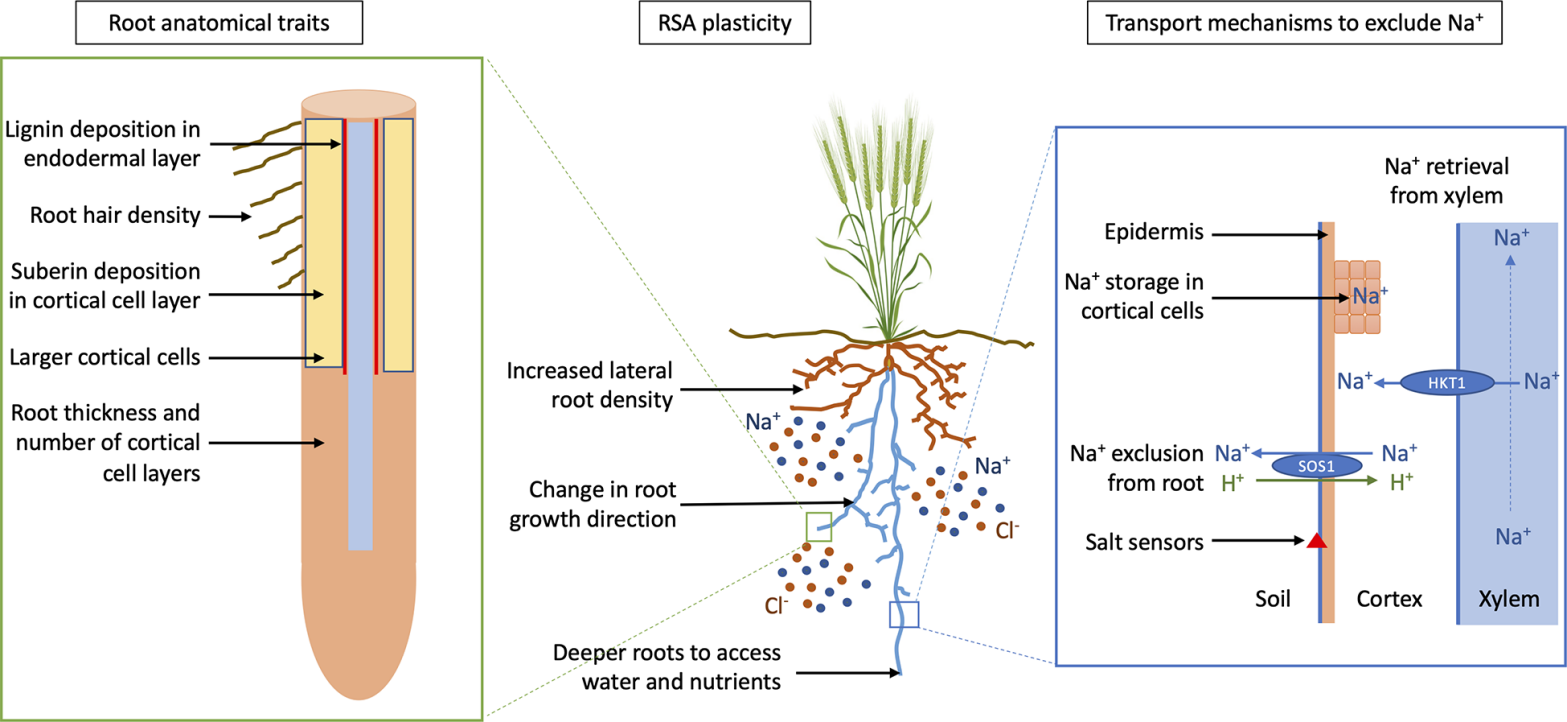
Crop root adaption for salt tolerance. Schematic diagram showing potential targets for designing a crop with a *salt-tolerant* root ideotype; *root anatomical traits* that prevent Na^+^ entry into the xylem and use less energy, *root system architecture* (RSA) plasticity to avoid salt whilst optimizing nutrient and water uptake, and *transport mechanisms for Na^+^ exclusion* from the root and retrieval from xylem, preventing accumulation of Na^+^ in the shoot. SOS1 (Salt Overly Sensitive 1) is a sodium/proton antiporter (Na^+^/H^+^) that facilitates export of Na^+^ out of the root and HKT1 (High affinity K Transporter 1) retrieves Na^+^ from the xylem. Adapted from “Wheat (grain filling stage)” by BioRender.com (2022).

The highly conserved salt overly sensitive (SOS) pathway, first discovered in Arabidopsis is undoubtedly critical for salt tolerance (Shi et al., 2000). The SOS pathway consists of SOS1, a plasma membrane localised Na^+^/H^+^ antiporter (**Figure 3**); SOS2, a CBL-interacting protein kinase (CIPK) family kinase, and SOS3, a calcineurinB-like (CBL) family Ca^2+^-binding protein. The SOS pathway is well characterised in Arabidopsis and is thought to confer salt tolerance by regulating net Na^+^ uptake at the soil-root interface. Although it has been extensively studied in Arabidopsis there is still relatively little known in crop plants. *HvSOS1* expression has been shown in the root stele and epidermis of barley roots (Zhu et al., 2016). In maize, a 4-bp frame-shifting deletion was identified in *ZmSOS1* impairing the function of SOS1, leading to a salt hypersensitive phenotype. A decrease in transcript level of *ZmCBL8* (a CBL/SOS3 component of maize SOS pathway) also led to increased shoot Na^+^ content under salt conditions, indicating that the SOS pathway is crucial for salt tolerance (Zhou et al., 2022).

## 5 Root system architecture

Root system architecture (RSA) refers to the spatial arrangement of the whole root system in the soil medium, modulated by root angle, type and rate of growth (Koevoets et al., 2016). The root surface area is also dependent on the development of root hairs and root diameter. RSA is complex and is considerably diverse among species, genotypes, and in space and time (Lynch, 1995). RSA is a plastic trait and responsible for establishing plant anchorage, optimising water and nutrient acquisition for plant growth, and avoiding unfavourable soil conditions (Smith and De Smet, 2012). Although there is significant research conducted on RSA in Arabidopsis, there is still limited research on the effect of salinity and other abiotic stresses on RSA in crop plants. One of the main limitations for understanding RSA is the difficulty in visualising root systems in soil.

### 5.1 Root phenotyping tools

Most research has been conducted on Arabidopsis using 2D systems such on rhizotrons, growth pouches, or transparent artificial growth media (agar). Non-destructive 2D high-throughput root phenotyping platforms include GrowScreen-Rhizo (Nagel et al., 2012), Phytomorph (Subramanian et al., 2013), GrowScreen-PaGe (Gioia et al., 2016), RADIX (Le Marié et al., 2016) and RhizoTubes (Jeudy et al., 2016) (reviewed in Atkinson et al., 2019). Phenotyping of crop RSA in response to salinity has included both non-soil based methods such as growing roots vertically on agar plates (barley, Arabidopsis) (Galvan-Ampudia et al., 2013; Shelden et al., 2013), pouches (maize, tomato) (Wang et al., 2020), paper rolls in PVC tubes (wheat) (Rahnama et al., 2011) and soil-based methods such as the clear pot method (wheat; sodicity) (Anzooman et al., 2019) and rhizotrons (tomato) (Gandullo et al., 2021) (**Table 1**). Wheat root systems have been extensively studied in the field using *shovel-omics* techniques (Ober et al., 2021).

**Table 1:** Arabidopsis and crop root phenotypic responses to salinity.

Species	Root Phenotype	NaCl concentration (mM)	Growth media	References
Arabidopsis	Primary root length decreased, and lateral root growth promoted	50	Agar	Zolla et al. (2010)
	Redistribution of biomass from main root to lateral roots	75 – 125	Agar	Julkowska et al. (2017)
	Halotropism – root bending away from salt	50 – 250	Agar	Galvan-Ampudia et al. (2013)
Barley	Reduction in number and length of seminal roots, reduced elongation rate	100 – 150	Agar	Shelden et al. (2013)
	Increased cortical cell length	100	Agar	Shelden et al. (2016)
	Apoplastic barriers in endodermis; suberin deposition in cortical cells in elongation zone	100	Agar	Ho et al. (2020)
	Reduced root hair density and root length	1 – 100	Hydroponics	Shabala et al. (2003)
Cotton	Primary root growth reduced above 75 mM, elongation rate decreased and shortened growth zone	1–150	Germination paper rolls	Zhong and Lauchli (1993)
	Length and weight enhanced of primary root, thinner roots	25 – 100	Hydroponics	Kurth et al. (1986)
Maize	Elongation rate reduced in the primary root and cell division inhibited	80	Hydroponics	Bernstein and Kafkafi (2002)
	Changes in RSA, reduced root growth	100	Soil - CT Scanning	Subramanian et al. (2015)
	Root biomass reduced; salt tolerance positively correlated with primary root depth	50 – 100	Semi-hydroponics	Wang et al. (2020)
Rice	Formation of apoplastic barriers	100 – 200	Hydroponics	Krishnamurthy et al. (2011)
	Lateral root density reduced in tolerant genotype	50 – 250	Hydroponics	Ijaz et al. (2019)
	Root biomass decreased, root: shoot biomass increased	50 – 150	Filter paper	Lin and Kao (1999)
	Shallow root growth angle	EC = 9 dS/m ^*^	Field (saline paddies)	Kitomi et al. (2020)
Rye	Cell division decreased and cell death increased	250	Hydroponics	Ogawa et al. (2006)
	Cell division increased	50 – 100	Hydroponics	Ogawa et al. (2006)
Sorghum	Halotropism – root bending away from salt	300 500	Agar Soil	Galvan-Ampudia et al. (2013)
	Root: shoot biomass increased	- 0.8 MPa ^*^	Hydroponics	Weimberg et al. (1984)
Tomato	Main root length decreased on agar and in pouches (not rhizotrons)	120	Rhizotrons, Pouches, Agar, Pots	Gandullo et al. (2021)
	Halotropism – root bending away from salt	300 500	Agar Soil	Galvan-Ampudia et al. (2013)
Wheat (Durum)	Seminal root growth inhibited	50 – 200	PVC tubes & filter paper	Rahnama et al. (2011)
	Reduction in biomass, root: shoot biomass increased	1 – 150	Pots (sand/ perlite)	Husain et al. (2004)
Wheat (Bread)	Seminal root growth decreased, lateral root growth enhanced, seminal root angle decreased	50 – 200	PVC tubes & filter paper	Rahnama et al. (2019)
	Narrower seminal root angle	ESP = 17 % ^*^	Clear pot method	Anzooman et al. (2019)
	Reduced root hair density and length	100	Hydroponics	Robin et al. (2016)
	Root biomass increased	EC < 9 ds/m ^*^	Soil	Nuttall et al. (2006)

* Electrical Conductivity (EC): 1 dS/m = 10 mM NaCl (Munns et al, 2020a); Osmotic Potential (MPa): - 0.1 MPa approximates 23 mM NaCl (Weimberg et al., 1984); Exchangeable Sodium Percentage (ESP) > 17 % = strongly sodic soil.

The development of non-destructive 3D phenotyping technologies such as X-ray computed tomography (CT), magnetic resonance imaging (MRI) and positron emission tomography (PET) (reviewed in Atkinson et al., 2019; Maqbool et al., 2022), will be extremely beneficial for future studies on root plasticity in crop species in response to environmental stresses such as salinity. X-ray CT scanning had been successfully employed to measure hydro-patterning in rice, maize and Arabidopsis (Bao et al., 2014) and salt stress in maize (Subramanian et al., 2015). To fully understand salt tolerance mechanisms in crops plants, measuring root traits non-invasively, and simultaneously with shoot traits will be crucial. Whole plant phenotyping capabilities such as the automated rhizotron platform GrowScreen-Rhizo provides the ability to simultaneous sense shoot and root growth (Nagel et al., 2012). 3D scanning can help to capture details of root systems such as lateral root growth and orientation that cannot be studied using traditional 2D methods. Differences in the structural complexity and space occupancy of the developing root system of corn seedlings in response to salinity has been reported using 3D images acquired from CT scanning (Subramanian et al., 2015). Improvements in root phenotyping platforms will enable screening for genetic diversity in crop species, allowing the selection of salt-tolerant genotypes that can be incorporated into breeding programs and evaluated in saline field conditions.

### 5.2 Changes in root system architecture in response to salinity

Many plants will exhibit changes in RSA in response to low soil moisture and salinity with the effect largely dependent on the type and severity of the stress, and the crop species. Salinity often occurs in arid and semi-arid lands, therefore crop plants have evolved mechanisms to cope with low water potential caused by salinity and drought (Munns and Tester, 2008). In wheat, the allocation of primary roots and lateral roots are strongly influenced by soil conditions including the soil moisture profile (Rich and Watt, 2013). A subsequent study found that wheat varieties developed for high yield on stored soil moisture have deep and vigorous root systems (Rich et al., 2016). This work was done in field soils in Australia and India, but most studies have been carried out in the laboratory in pots filled with soil or on media with artificial substrates (**Table 1**). In the next two sections, we compare studies evaluating changes in RSA in response to salinity conducted using non-soil-based methods and pot studies.

In both barley and wheat, seminal root growth was inhibited by NaCl concentrations in the root medium in a dose dependent manner (Rahnama et al., 2011; Shelden et al., 2013). In a study on bread wheat (*Triticum aestivum* L.), root growth and branching patterns were modified in response to salinity with primary root growth inhibited and branch root elongation promoted (Rahnama et al., 2019). In durum wheat (*Triticum turgidum* L. ssp *durum*), seminal root growth was inhibited more than branch roots in three out of four genotypes examined (Rahnama et al., 2011).

In Arabidopsis, primary and lateral root length, and lateral root number, were consistently reduced due to salinity stress (Karlova et al., 2021). Salt stress reduced primary root length more severely than elongation of lateral roots (Julkowska et al., 2017), and lateral root growth emergence was promoted in response to salt stress (Zolla et al., 2010; Galvan-Ampudia and Testerink, 2011). Natural variation in RSA has been observed in Arabidopsis accessions, which was linked to the expression of HKT1 (a transporter that removes Na^+^ from the xylem) in the roots (Julkowska et al., 2017). Also in Arabidopsis, it was observed that emerging and young lateral roots were less likely than primary roots to undergo programmed cell death at the lethal salt concentration of 200 mM NaCl (Ambastha et al., 2020).

In the tolerant rice genotype KS-282, the lateral root density was reduced in response to salt stress, compared to the sensitive genotype (Ijaz et al., 2019). In tomato, phenotypic analysis using rhizotrons showed that the distribution of lateral roots changed in response to salt stress with lateral root formation inhibited primarily at the soil surface (Gandullo et al., 2021). Overall, these observations suggest that the pattern of root system architecture might change in favour of deeper roots with fewer lateral roots, maximising the opportunity to access deeper and less saline water tables. However, the total root mass is still reduced in comparison to plants in non-saline soils.

### 5.3 Field studies

Phenotyping root traits in the field, although possible, is constrained by laborious sampling methods that are time-consuming, costly and require significant inputs of labour. This is usually done when crops reach maturity as sampling in the field is destructive and done at the same time as harvest. Field sites can also experience yearly variations due to changing environmental conditions, influencing the mature root systems (Rich et al., 2016). High-throughout, automated and affordable field phenotyping is still in development (Rich et al., 2016; Wasson et al., 2016). Wasson et al. (2016) developed a portable fluorescence imaging system (BlueBox) to automate root counting in soil cores with image analysis software directly in the field. Controlled-environment screens are aimed at circumventing the problems associated with field phenotyping, however these mostly focus on seedling and immature plants due to constraints with pot size and glasshouse facilities. Given this, there are relatively few studies that measure root traits in saline soils in field conditions.

Soil cores taken from the field and placed in pots in controlled environment conditions provide a representative experimental system. Plants were grown in cores from saline soils and compared to cores taken from benign soils (Nuttall et al., 2006). Root growth of durum wheat and lentil (both moderately salt-sensitive species) was reduced at depths of 0.3 m in the hostile soil. The root system of barley adapted so that root biomass was greater in soil to a depth of 0.4 m, however at deeper positions in the soil profile there were fewer roots resulting in less extraction of water at depth.

Sodicity is a common occurrence in saline soils. Using the clear pot method, seedling coleoptile length and root angle were measured in different bread wheat genotypes grown in sodic soils (Richard et al., 2015; Anzooman et al., 2019). Genetic variation in seminal root angle was observed, with genotypes that had a narrower root angle having greater root depth, potentially allowing the roots to obtain soil moisture from deeper layers.

In rice, the *qSOR1* gene (responsible for the surface rooting phenotype) was identified to be a *DRO1* homolog in rice (deep rooting allele) and appears to be involved in the root gravitropic response (Kitomi et al., 2020). In saline paddies, near-isogenic lines carrying the qSOR1 loss-of-function allele had soil-surface roots (SOR) that enabled rice to avoid the reducing stresses of saline soils, resulting in increased yields compared to the parental cultivars without SOR.

Although both salt and drought stress result in a water deficit stress around the roots, changes to RSA are quite distinct between the two stresses (Viana et al., 2022). The root response may be dependent on how the stress is imposed, for example, water stress induced by withholding water from pots filled with clay-based soil will result in soil compaction, whereas use of a substrate like vermiculite (Yamaguchi and Sharp, 2010) or perlite avoids this. Salinity stress is usually applied in hydroponics or sand culture and can be kept at a constant degree. Drought is unavoidably a gradual change over time; the use of liquid media containing osmolytes is not recommended. The addition of NaCl will impose an initial osmotic stress. Primary root growth is often maintained in response to both water deficit and salinity and root: shoot ratio is increased. In maize, genotypes with fewer but longer lateral roots had increased rooting depth (Zhan et al., 2015). In contrast, in Arabidopsis, there was a redistribution of biomass from the main root to lateral roots (Julkowska et al., 2017).

## 6 Halotropism: A strategy to avoid salt

Gravitropism is central to root growth as it is the gravity stimulus that drives the direction of root growth, with gravity sensing known to occur in the columella cells in the root tip. Salinity can interfere with the gravitropic response. Negative halotropism is the response of roots to grow away from salt. This could be a trait that is specific for salt-sensitive species, since in the halophytes (salt-tolerant species) *Bassia indica* and *Limonium bicolor*, the opposite phenotype – where roots grow towards salt, termed “positive halotropism”, has been observed (Shelef et al., 2010; Leng et al., 2019). Whether halotropism exists in crops, rather than root growth being inhibited by decreased water potential of the soil (due to drought or salinity), is unknown, and the costs to the plant modifying root architecture are unexplored (Munns and Gilliham, 2015). For roots to grow away from salt at depth, the exposure to salt must challenge or modify the plants response to gravity, causing the root growth direction to change. In the salt-sensitive species, tomato and sorghum, root bending away from salt is a strategy to avoid stress (Galvan-Ampudia et al., 2013). Root bending was only seen at > 300 mM NaCl concentrations indicating that the salt concentration needs to be relatively high to induce a halotropic root response.

Much of the research conducted on halotropism and root system architecture traits have been in the model plant, Arabidopsis. In Arabidopsis, the negative halotropic response was shown to be salt-specific ie. the roots did not change direction due to an equivalent osmotic gradient (induced by mannitol or KCl). The molecular mechanism for halotropism is due to the redistribution of the hormone auxin through the regulation of the auxin protein PIN2 (Galvan-Ampudia et al., 2013). Halotropism is dependent on the asymmetric redistribution of the hormone auxin from the side of the root closest to the salt, to the side of the root furthest from the salt. The salt-specific response involves changes in expression and localisation of the two auxin transporters, PIN2 (influx) and AUX1 (efflux) in the root tip (van den Berg et al., 2016). Arabidopsis plants overexpressing SOS1, showed a reduced halotropic response and Arabidopsis *sos1* and *sos2* mutants showed halotropic responses at lower thresholds of NaCl (Galvan-Ampudia et al., 2013). Taken together, these observations provide evidence that high internal concentrations of cellular Na^+^ may trigger the directional root growth, however, this has been unexplored experimentally. Differences in Na^+^ sequestration in the roots of durum and bread wheat, have been linked with differences in salt tolerance, however it is unknown if internal salt concentrations correlate with the halotropic response (Wu et al., 2018a). Whether other crop species, such as barley and wheat, have similar halotropic responses and share the same regulatory mechanisms is unknown.

Soil salinity, in both natural and managed environments (rain-fed and irrigated), is highly heterogeneous, and understanding how plants respond to this spatial and temporal heterogeneity is important for sustainable agriculture in more marginal lands. Split-root studies have shown how plants compensate for water and or nutrient deficiency, by optimizing root-foraging in the most favourable parts of the soil. This also applies to a saline soil. A review of halophytes grown with roots split between low and high salinity media, versus uniformly saline media, showed that water uptake from the least saline part of the soil or solution was the key factor driving shoot growth. Plants with part of the root at low salinity (0–10 mM NaCl) had 3- to 10-fold higher shoot dry mass than plants with roots in uniformly saline (50–800 mM NaCl) media (Bazihizina et al., 2012).

Plants exclude 97-98% of the salt, as remarked in the Introduction, so unless the soil receives frequent rainfall, or irrigation, the salt will build up to concentrations that prevent uptake further uptake of water. This implies that roots in saline soils without irrigation must continually forage for low-salt patches while balancing the need for nutrients. The root system architecture must be flexible enough to continue *in situ* if there is a history or ‘memory’ of frequent rain or irrigation that dilutes the salt that builds up around the roots or continue the exploration for low salt patches. The latter could become very expensive in terms of carbon cost.

## 7 Salinity induces root anatomical changes

Apoplastic barriers such as suberin lamellae and casparian bands are thought to limit the movement of salt transversely across the root by mass flow. In barley, salt stress reduces the hydraulic conductivity in the system by reducing water flow along the transcellular path (Knipfer et al., 2021). Transcriptomic and metabolomic analyses showed over-representation of genes and metabolites involved in the phenylpropanoid biosynthesis pathway in barley roots (Ho et al., 2020). Genetic differences have been observed in the formation of apoplastic barriers in barley genotypes (Ho et al., 2020) and rice (Krishnamurthy et al., 2011). Salt stress resulted in an increase in the production and abundance of apoplastic barriers in the endodermis of the Australian domesticated cultivar Clipper (Ho et al., 2020). In contrast, high levels of suberin synthesis-related gene expression and localization of suberin in cortical cells was observed in the elongation zone of the landrace Sahara, most likely restricting apoplastic transport of Na^+^ (**Figure 3**). At 100 mM NaCl, Clipper accumulated higher concentrations of Na^+^ in the root maturation zone compared to the landrace Sahara (Shelden et al., 2013; Shelden et al., 2020). In both genotypes, K^+^ strongly correlated with Na^+^ concentration. A similar result was found in a transcript and proteomics analysis by Yamaguchi and Sharp (2010) with maize roots grown at low water potentials in vermiculite (low water content), suggesting that the formation of apoplastic barriers due to suberin and lignin deposition in cell walls of the stele was a response to low water potential. The deposition of suberised barriers in rice roots correlated with a sharp reduction in the uptake of Na^+^ in the shoot (Krishnamurthy et al., 2011).

An increase in cortical cell diameter was shown to occur in response to drought in wheat and is believed to lead to a reduction in energy usage (Colombi et al., 2019). Decreasing the number of cortical cell files by one or two can also reduce the energy costs associated with Na^+^ exclusion (Foster and Miklavcic, 2019; Munns et al., 2020a). Enlargement of root cortical cells in response to salt stress has been reported in barley (Shelden et al., 2016), maize (Li et al., 2014), cotton (Kurth et al., 1986) and halophytes (Hajibagheri et al., 1985) and may be due to increased vacuolation. Vacuolation is an adaptive mechanism to facilitate osmotic adjustment by the compartmentalisation of Na^+^ and Cl^-^.

Plasticity in the development of root epidermal cells and differentiation of root hairs is thought to be a response to sensing environmental signals and adjusting to the stress response. In Arabidopsis, root hair density and length decreased in a dose dependant manner in primary roots (Wang et al., 2008). In both barley and wheat, root hair density and length were significantly reduced by salinity (Shabala et al., 2003; Robin et al., 2016), however osmotic stress has been shown to increase root hair density (Robin et al., 2021). Munns et al. (2020a) speculated that root hair cells may sequester salt into vesicles that are moved to the cell surface and extruded into the soil like what occurs in epidermal bladder cells of halophytes.

## 8 Root tissue-specific expression of salt and water transporters

Membrane transporters located in the root tissues are responsible for the uptake of both Na^+^ and Cl^-^ ions from the soil, and for the uptake of water by aquaporins (Chaumont and Tyerman, 2014). Understanding the spatial and temporal expression of salt and water transporters is important to gain a complete understanding of salinity tolerance and root adaptation. It is also necessary to gain insights into how we can engineer crops to have improved root systems adapted to specific environments. At any point in time there are changes in the cell-specific spatial response to stress. Our ability to measure these changes are now enhanced by having access to high resolution ‘omic technologies for spatial profiling of metabolites, gene transcripts, proteins and ions (Shelden and Roessner, 2013).

The spatial and temporal variation in the expression of different transporters involved in water and nutrient transport in crop root tissues has been previously reported by Arsova et al. (2020), however our knowledge in this area is still limited, especially in crop plants. Salt overly sensitive (SOS1), a Na^+^/H^+^ antiporter, has previously been shown to be expressed in the epidermal cells surrounding the root apex in Arabidopsis (Shi et al., 2002) and barley (Zhu et al., 2016). Root epidermal cells contain Na^+^ transporters such as HvHKT1;1 in barley (Han et al., 2018). The distribution of membrane transporters associated with salt and water transport varies with both root age and space however mapping of transporters is biased towards Arabidopsis and young roots as information is lacking from other species and older roots (Arsova et al., 2020).

There are several genes encoding membrane transporters that have proven potential for increasing salt tolerance in crops. These include *Nax2* from wheat (Munns et al., 2012), *SKC1* (*OsHKT1;5*) from rice (Ismail and Horie, 2017) and the HAK family in maize seedlings (Zhang et al., 2019). Na^+^ exclusion genes, *Nax1* (*TmHKT1;4-A2)* and *Nax2* (*TmHKT1;5-A)* from the diploid wheat ancestor *Triticum monococcum* were introgressed into durum wheat Tamaroi and have both been shown to remove Na^+^ from the xylem in roots (Munns et al., 2012). *Nax2* is expressed in the stele, specifically in the plasma membrane of the parenchyma cells lining the xylem vessels (Munns et al., 2012) (**Figure 3**). *Nax1* can also remove Na^+^ from the xylem flowing through leaf sheaths (James et al., 2011). *Nax2* in Tamaroi resulted in higher grain yields in response to both salinity (Munns et al., 2012) and sodicity (Genc et al., 2016). *TaHKT1;5-D Kna1* locus is localised on the plasma membrane of the root stele of bread wheat (*Triticum aestivum* L.) and retrieves Na^+^ from the xylem (Byrt et al., 2014).

## 9 Future perspectives: Breeding crops with root traits for salt-tolerance

Whilst there is a significant amount of research on root adaptation to salinity in the model plant Arabidopsis, we now need to focus our efforts on expanding our knowledge in crop species. Many of our major crop species are monocotyledons (ie. wheat, barley, rice, and maize) and have different root types and anatomical features compared to the dicot Arabidopsis. It is therefore likely they will have different mechanisms to adapt to saline soils. Understanding the root response to salt stress is complicated as it is dependent on species, genotype, plant age, and the intensity and duration of the stress. Many root studies have also been carried out in the laboratory in pots filled with soil or on media with artificial substrates, with very limited field studies. The advent of new high-throughput phenotyping platforms and technologies will help us to gain a better understanding of the diversity of root responses to saline soils across species. In the future, it will also be imperative to assess root traits in the field, and capture root responses to heterogenous soils.

Exploiting root traits with specific ideotypes in crop breeding has been shown to be successful for nitrogen acquisition and water capture in maize (Lynch, 2019). Salinity is a multigenic trait and identifying a specific root ideotype for salinity tolerance will be more challenging. We propose a *salt-tolerant* root ideotype for designing a root system adapted to saline soils. The *salt-tolerant* root ideotype would include root plasticity to allow roots to avoid highly saline soils whilst maximising water and nutrient uptake, transport mechanisms to exclude Na^+^ from the root and root anatomical traits to restrict Na^+^ movement into the xylem and conserve energy (**Figure 3**). The identification of specific root traits adapted to saline soils and with current advances in crop genetics, these traits can be incorporated into crop breeding programs, leading to improved yields on saline soils.

## Author contributions

MS and RM both contributed to writing the article. All authors contributed to the article and approved the submitted version.
